# Fluconazole-Resistant *Candida parapsilosis* Bloodstream Isolates with Y132F Mutation in *ERG11* Gene, South Korea 

**DOI:** 10.3201/eid2409.180625

**Published:** 2018-09

**Authors:** Yong Jun Choi, Yae-Jean Kim, Dongeun Yong, Jung-Hyun Byun, Taek Soo Kim, Yun Sil Chang, Min Ji Choi, Seung Ah Byeon, Eun Jeong Won, Soo Hyun Kim, Myung Geun Shin, Jong Hee Shin

**Affiliations:** Chonnam National University Medical School, Gwangju, South Korea (Y.J. Choi, M.J. Choi, S.A. Byeon, E.J. Won, S.H. Kim, M.G. Shin, J.H. Shin);; Samsung Medical Center, Sungkyunkwan University School of Medicine, Seoul, South Korea (Y.-J. Kim, Y.S. Chang);; Yonsei University College of Medicine, Seoul (D. Yong, J.-H. Byun);; Seoul National University Hospital, Seoul (T.S. Kim)

**Keywords:** Candida parapsilosis, fluconazole, drug resistance, fungi, microsatellite marker, candidemia, antimicrobial resistance, South Korea

## Abstract

We recently observed the emergence of fluconazole-resistant *Candida parapsilosis* bloodstream isolates harboring a Y132F substitution in Erg11p in South Korea. These Y132F isolates had a higher propensity to cause clonal transmission than other fluconazole-resistant isolates and persisted within hospitals for several years, as revealed by microsatellite typing.

*Candida parapsilosis* is the second most common species isolated from patients with *Candida* bloodstream infections (BSIs) in Latin America and eastern Asia (*1**,**2*). Although uncommon, fluconazole-resistant *C. parapsilosis* isolates harboring the Y132F substitution in Erg11p (referred to as Y132F isolates) have been reported in Brazil, the United States, and Kuwait (*3**–**6*). The precise reason for the emergence of *C. parapsilosis* Y132F isolates has yet to be defined; it may be related to selective drug pressure, and the mutation at position 132 may be a hot spot for resistance mediated by *ERG11*, a gene encoding the azole target (*3*). Alternatively, *C. parapsilosis* Y132F isolate emergence may be associated with exogenous clonal transmission (*4*). We recently observed the emergence and nosocomial spread of Y132F isolates in South Korea. In this study, we report a greater increase in the clonal spread of *C. parapsilosis* Y132F BSI isolates than of non-Y132F fluconazole-resistant isolates within hospitals during the past several years.

We assessed the first 47 *C. parapsilosis* BSI isolates that were fluconazole-resistant (MIC ≥8 mg/L) according to the Clinical and Laboratory Standards Institute (CLSI) species-specific clinical breakpoint (*7**,**8*). All 47 isolates were obtained from multicenter surveillance cultures from 8 university hospitals (A–H) during 2005–2016. For all fluconazole-resistant isolates, we examined genotypic relationships using microsatellite typing. We defined >2 isolates with identical genotypes according to microsatellite typing as clonal isolates. We sequenced the *ERG11* gene and 3 transcription factor genes: *TAC1*, which can lead to the upregulation of *CDR*; *MRR1*, which can lead to the upregulation of MDR; and *UPC2*, which can lead to the upregulation of *ERG11* (*5*); we compared the results with those of 20 fluconazole-susceptible (MIC 0.5–2 mg/L) isolates. This study was approved by the Institutional Review Board of Chonnam National University Hospital (IRB CNUH-2014-290).

Of the 47 *C. parapsilosis* fluconazole-resistant isolates, 30 (63.8%) had the Y132F substitution in Erg11p; however, none of the 20 fluconazole-susceptible isolates had the Y132F mutation in *ERG11*. Recently, 31%–57% of fluconazole-resistant *C. parapsilosis* isolates from different parts of the world were reported to be Y132F isolates, but the Y132F mutation was absent in all fluconazole-susceptible isolates (*3**–**6*). These data confirm that a Y132F substitution in Erg11p is the predominant fluconazole resistance mechanism for *C. parapsilosis* worldwide.

Microsatellite typing revealed that 4 clonal Y132F isolates (M1–4) were persistently recovered in 2 hospitals (A and B) over a period of 3–7 years, and the proportion of clonal isolates was much higher in Y132F isolates (86.7%, 26/30) than in non-Y132F fluconazole-resistant isolates (11.8%, 2/17) (Table). In a previous microsatellite study from a US surveillance study by Grossman et al. (*4*), no hospital specificity was detected among 13 non-Y132F fluconazole-resistant isolates; however, 2 notable clusters of isolates from 17 Y132F isolates were found over 8- or 18-month periods. The results obtained in our study and those of Grossman et al. indicate that Y132F isolates may have a higher propensity to cause clonal transmission and persist in particular hospitals than do non-Y132F fluconazole-resistant isolates. The Y132F substitution in Erg11p has also been detected in *C. auris* isolates from Pakistan (10/16 isolates), India (12/17 isolates), and Venezuela (5/5 isolates); these isolates are strongly associated with clonal transmission (*9*). Further studies are needed to determine whether the Y132F mutation in Erg11p has a direct effect on clonal transmission of *C. parapsilosis* or *C. auris* isolates.

**Table:** Molecular characterization of 47 fluconazole-resistant isolates and 20 fluconazole-susceptible isolates of *Candida parapsilosis*, South Korea*

Microsatellite genotypes†	Hospital	No. isolates	MICs, mg/L‡			Amino acid substitutions§				Isolation year (no. patients)
FLC	VRC	Erg11p	Mrr1p	Tac1p	Upc2p
Fluconazole-resistant with Y132F in Erg11p, n = 30 isolates										
M1	A	8	8–32	0.25–0.5		Y132F	K177N			2006 (1), 2009 (1), 2010 (2), 2011 (2), 2012 (1), 2013 (1)
	B	3	16–32	0.5		Y132F	K177N			2012 (2), 2013 (1)
M2	A	10	8–32	0.125–0.5		Y132F	K177N			2012 (1), 2016 (9)
M3	A	3	8–16	0.25		Y132F	K177N, Q1053*			2007 (1), 2011 (1), 2012 (1)
M4	A	2	8–>64	0.5–4		Y132F	K177N			2013 (1), 2016 (1)
M5	A	1	32	0.25		Y132F	K177N			2012 (1)
M6	A	1	8	0.5		Y132F	K177N			2013 (1)
M7	A	1	8	0.25		Y132F	K177N			2016 (1)
M8	C	1	64	2		Y132F				2016 (1)
Other fluconazole-resistant, n = 17 isolates										
M9	D	2	>64	1			G583R			2007 (1), 2009 (1)
M10	E	1	16	0.5		R398I		L877P		2005 (1)
M11	A	1	8	0.25						2006 (1)
M12	E	1	8	0.25		R398I		L877P		2011 (1)
M13	F	1	16	0.06		R398I		L877P		2011 (1)
M14	E	1	8	0.125		R398I		L877P		2012 (1)
M15	G	1	8	0.125		R398I		L877P		2012 (1)
M16	G	1	8	0.06		R398I		L877P		2012 (1)
M17	E	1	8	0.125				N900D		2012 (1)
M18	C	1	8	0.125		R398I	P250S	L877P		2012 (1)
M19	C	1	8	0.25		R398I	S1081P	L877P		2012 (1)
M20	D	1	8	0.125		R398I			D394N	2012 (1)
M21	E	1	32	0.5		R398I	P295R	L877P		2015 (1)
M22	E	1	16	0.125		R398I				2015 (1)
M23	H	1	32	0.125		K128N	W872C			2015 (1)
M24	E	1	16	0.25			G927D			2016 (1)
Fluconazole-susceptible controls										
M3	A	1	1	0.03			K177N, Q1053*			2010 (1)
M25	C	2	0.5	0.03		R398I				2012 (2)
M26	F	2	0.5	0.03–0.06				R208G		2012 (1), 2013 (1)
M27	A	1	2	0.06						2010 (1)
M28	A	1	0.5	0.03			K177N, Q1053*			2011 (1)
M29	A	1	1	0.06				L877P		2011 (1)
M30	A	1	1	0.03				R208G		2012 (1)
M31	A	1	0.5	0.03				R208G		2012 (1)
M32	E	1	2	0.03						2012 (1)
M33	G	1	0.5	0.03				R208G		2012 (1)
M34	G	1	0.5	0.03						2012 (1)
M35	A	1	0.5	0.03				R208G		2013 (1)
M36	A	1	1	0.06		R398I			D394N	2013 (1)
M37	A	1	0.5	0.03		R398I				2013 (1)
M38	D	1	0.5	0.06		R398I				2014 (1)
M39	D	1	0.5	0.06				R208G		2014 (1)
M40	E	1	0.5	0.03				R208G		2015 (1)
M41	A	1	1	0.03		R398I		L877P		2015 (1)

*CLSI, Clinical and Laboratory Standards Institute; FLC, fluconazole; VRC, voriconazole. †For microsatellite typing, each strain was characterized by a genotype resulting from combination of the sizes of the 4 markers (CP1, CP4, CP6, and B). See the Technical Appendix Figure for results of microsatellite genotyping presented as an UPGMA tree. ‡Antifungal MICs were determined by the CLSI M27–A3 broth microdilution method (*7*). The fluconazole MICs of 30 Y132F isolates determined by Etest were ≥8 mg/L. All 67 isolates tested were susceptible to amphotericin B (MIC 0.25–1 mg/L) and micafungin (MIC 0.25–2 mg/L) according to the CLSI method. §All were homozygote alleles except for 6 heterozygote alleles (Q1053,* G583R, P250S, P295R, W872C, and G927D) in Mrr1p.

Two previous studies conducted in the United States detected the Erg11p Y132F substitution in combination with the Erg11p R398I substitution in almost all *C. parapsilosis* BSI isolates (*4**,**5*). In addition, no Y132F isolates detected in a US surveillance study contained an *MRR1* polymorphism, according to *MRR1* sequence analysis results (*4*). However, a single Y132F substitution in Erg11p was found in all 30 fluconazole-resistant isolates from South Korea hospitals. The same K177N substitution in Mrr1p was found in all Y132F isolates except 1; none of the Y132F isolates showed missense mutations in Tac1p or Upc2p (Table). Taken together, these findings demonstrate low genetic diversity among Y132F isolates from the same country (the United States or South Korea).

In our study, 76.7% (23/30) of patients with Y132F isolates had no antifungal exposure within 30 days before candidemia detection, and their clonal transmission was not detected by routine hospital surveillance, partly because more than half of the patient hospitalizations did not overlap. These findings indicate that clonal Y132F isolates may be dormant over long periods and can survive and persist outside their host on hospital environmental surfaces, which may be similar to the behavior of *C. auris* (*10*). Although our study was limited by the relatively low number of isolates, our data suggest that *C. parapsilosis* Y132F isolates should be identified in clinical microbiology laboratories to prevent further clonal transmission of BSI caused by Y132F isolates.

Technical Appendix Results of microsatellite typing presented as an UPGMA tree.
