# Supplementary material for: Fluconazole-Resistant Candida parapsilosis Bloodstream Isolates with Y132F Mutation in ERG11 Gene, South Korea
Source: Emerg Infect Dis. 2018 Sep;24(9):1768–70. doi: 10.3201/eid2409.180625 (PMC6106402; doi:10.3201/eid2409.180625)
Supplement: Technical Appendix — Results of microsatellite typing presented as an UPGMA tree. [file 18-0625-Techapp-s1.pdf]

Article DOI: https://doi.org/10.3201/eid2409.180625

Fluconazole-Resistant *Candida parapsilosis* Bloodstream Isolates with Y132F Mutation in *ERG11* Gene, South Korea

Technical Appendix

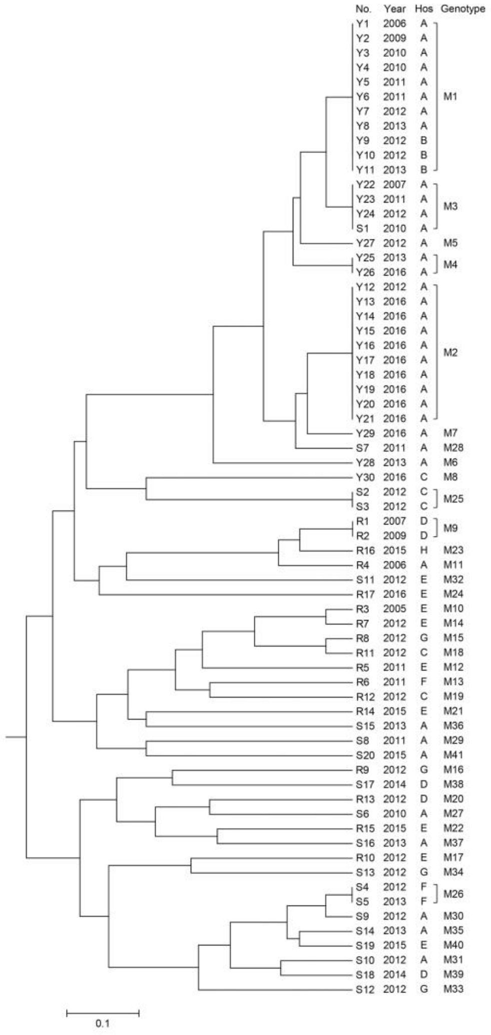

Technical Appendix Figure. Results of microsatellite genotyping presented as an unweighted pair group method with arithmetic mean (UPGMA) tree for 67 bloodstream isolates of *Candida parapsilosis* from South Korea. No., numbers of 30 Y132F isolates (Y1–Y30), other 17 fluconazole-resistant isolates (R1–R17), and 20 fluconazole-susceptible isolates (S1–S20); Hos, hospital.
